# The interplay between diabetes Mellitus and soft tissue infections in general surgical patients

**DOI:** 10.1186/s12902-024-01636-y

**Published:** 2024-07-08

**Authors:** Stephanie Cheng, Benjamin Rui-Min Poh, Vivyan Wei Yen Tay, Piea Peng Lee, Sachin Mathur

**Affiliations:** 1 Department of General Surgery, Singapore General Hospital, Singapore, Singapore; 2 Division of Surgery and Surgical Oncology, National Cancer Centre, Singapore, Singapore

**Keywords:** Abscess, Acute care surgery, Diabetes, Infection, Soft tissue infections

## Abstract

**Background:**

Diabetes mellitus (DM) is a worldwide pandemic affecting 500 million people. It is known to be associated with increased susceptibility to soft tissue infections (STI). Despite being a major public health burden, the literature relating the effects of DM and the presentation, severity and healing of STIs in general surgical patients remain limited.

**Method:**

We conducted a retrospective review of all patients admitted with STI in a tertiary teaching hospital over a 12-month period. Patient demographics and surgical outcomes were collected and analysed.

**Results:**

During the study period, 1059 patients were admitted for STIs (88% required surgery). DM was an independent risk factor for LOS. Diabetic patients presented with higher body-mass index (28 vs. 26), larger abscess size (24 vs. 14 cm^2^) and had a longer length of stay (4.4 days vs. 2.9 days). They also underwent a higher proportion of wide debridement and application of negative pressure wound therapy (42% vs. 35%). More diabetic patients underwent subsequent re-operation within the same sitting (8 vs. 4). Diabetic patients were two times more likely to present with carbuncles (*p* = 0.02).

**Conclusion:**

The incidence of STIs among DM patients represent a significant disease burden, surgeons should consider intensive patient counselling and partnering with primary care providers in order to help reduce the incidence of future STI admissions based upon lifestyle modification and glucose control.

## Introduction

Diabetes mellitus (DM) is a global pandemic affecting the lives of 500 million people [1]. The prevalence is expected to rise 69% between 2010 and 2030 in developed countries [2]. The disease is characterised by accelerated atherosclerosis with resultant micro- and macrovascular complications including retinopathy, nephropathy and peripheral vascular disease (PVD) [3]. DM is associated with reduced cell-mediated, T cell and neutrophil responses, as well as disorders of humoral immunity [4]. The susceptibility to soft tissue infections (STI) may affect between 20 and 50% of DM patients [5]. A large retrospective cohort study showed at least a 2-fold risk in STI’s amongst diabetic patients compared to non-diabetic controls [6]. The major health burden of such common infections affecting general practice, outpatient clinics and in-patient hospital care cannot be underestimated.

STI’s may be classified according to anatomic location, causative pathogen, depth of infection and severity of clinical presentation [7]. Wide patterns of presentation include simple subcutaneous abscess or cellulitis vs. deep seated infections requiring debridement and delayed wound closure. These conditions are core practice for the emergency general surgeon and decisions regarding extent and timing of surgery are critical to patient outcomes. Regardless of type of infection, patients with DM suffer a 2-fold increase in mortality [8].

Despite most evidence being limited to observational and case series, strong recommendations regarding early intervention and source control remain the standard management of soft tissue infections in surgical patients [9]. However, studies in the surgical literature pertaining to the effect of DM on presentation, severity and healing of soft tissue infections remain limited. In the current study, we hypothesized that diabetic and non-diabetic patients who present with soft tissue infections have different clinical outcomes, including morbidity, Length of stay (LOS), negative pressure wound therapy (NWPT) usage, re-admission, re-operation and abscess recurrence. We aim to describe the relationship between DM and STI’s in a general surgical cohort.

## Methods

### Study design

A retrospective review was undertaken of all patients admitted under the Department of General Surgery in a tertiary public hospital with STI over a 12-month period. Data on patient demographics including age, body-mass index (BMI), comorbidities, site, laboratory investigations results, size of abscess, American Society of Anaesthesiologists (ASA) grade, LOS (LOS), surgery type and clinical outcomes were collected. The size of the abscess was measured by length (cm) x breadth (cm) of the abscess in medical records. The area of abscess was estimated by length multiplied by breadth. STI was classified as uncomplicated (cellulitis/soft tissue abscess) or complicated (carbuncle/necrotizing infection). All diabetic (Type 1/2) patients were included in the study.

In both groups antibiotic treatment according to institutional guidelines was given systematically once diagnosis was established. Qualified surgeons performed incision and drainage (I&D) or wide debridement in the operating theatre setting, usually under general anaesthesia (GA). Patients were clean and draped, elliptical incision made over abscess to allow sufficient for drainage for cases requiring I&D; infected tissue debrided until healthy tissue seen in cases requiring wide debridement. All wounds are left open to heal with secondary intention, with or without the use of NPWT.

The need for NPWT usage is a joint decision made by a multidisciplinary team comprising of surgeons and specialty wound nurses.

### Exclusion criteria

Patients that presented with limb and diabetic foot abscesses were excluded as they fell under the care of Orthopaedic/Vascular Surgery.

### Outcome measures

The primary outcome was type and size of infection at presentation. The secondary outcomes included LOS, need for reoperation, readmission within 30 days and recurrence within 6 months.

### Statistical analysis

In the descriptive analysis of patient characteristics, summary statistics were applied as appropriate. These were examined and compared between diabetic and non-diabetic patients. For continuous variables, we reported means, SDs, medians, minimum and maximum values and interquartile ranges. For categorical variables, absolute (n) and relative (%) frequencies were reported. We used proportion tests as well as independent two samples t-tests and median to compare means and medians across the two different groups.

For regression analyses, we randomly ordered the dataset, split the first 80% into a training set and the last 20% into a test set, and performed univariate as well as two types of multivariable multinomial logistic regression [10–12] models to estimate the odds ratios. Robust regression was used to model the LOS as the distribution of LOS showed outliers or influential observations. To construct the multivariable models, we chose independent variables for each outcome, resulting in two model types as follows: 1) a full model with all independent variables that were not correlated with each other; and (2) a model that included only independent variables that were not correlated with each other, but statistically significant in their univariate analyses where the p values are smaller than 0.5. We also used accuracy calculated from the classification table, residual standard errors and AUC-ROC score to determine model fit of multivariable regression analyses.

The data was strictly confidential and anonymous. This study of deidentified data was approved by the institutional review board. All statistical analyses were performed using R Studio (version 2023.06.0 Build 421) and R Statistical Software (version 4.3.1; R Foundation for Statistical Computing, Vienna, Austria) with a p-value of < 0.05 considered statistically significant.

## Results

During the study period, 1059 patients were admitted for STI and the demographics are shown in Table 1.

**Table 1:** Demographics

Characteristics	Number of patients (%)		
Total no of patients	**1059**		
No. of patients that underwent surgery	**936 (88.3)**		
No. of patients that did not undergo surgery - Conservative management - AOR discharge - Bedside I&D	**123 (11.6)** 106 11 6		
Male	672 (63.4)		
Median age (range)	48 (15–98)		
Above 65 years old	190 (17.9)		
Smoker	790 (74.6)		
Median BMI (range)	26.2 (14.4–73)		
Patients with DM of which newly diagnosed at admission	330 (31.3) 29 (2.7)		
Median LOS (days, range)	3 (1–87)		
Recurrence of abscess after 6 months	84 (7.9)		
	**DM (%)** ** (** ***n*** ** = 330)**	**Non-DM (%)** ** (** ***n*** ** = 729)**	**Total (%)**
**ASA** ** (** ***n*** ** = 1059)**			
1	6 (1.81)	235 (32.3)	241 (22.8)
2	161 (48.8)	396 (54.3)	557 (52.6)
3	160 (48.5)	94 (12.9)	254 (24.0)
4	3 (0.9)	4 (0.5)	7 (0.66)
**Type of infection** ** (** ***n*** ** = 1059)**			
Cellulitis (uncomplicated)	12 (3.63)	31 (4.2)	43 (4.06)
Abscess (uncomplicated)	272 (82.4)	650 (89.2)	922 (87.1)
Carbuncle (complicated)	45 (13.6)	48 (6.6)	93 (8.78)
Necrotizing fasciitis (complicated)	1 (0.3)	0 (0)	1 (0.09)
**Site of abscess** ** (** ***n*** ** = 1059)**			
Head and neck	36 (10.9)	41 (5.6)	77 (7.27)
Axilla	13 (3.9)	60 (8.2)	73 (6.89)
Groin	16 (4.8)	40 (5.5)	56 (5.57)
Back	41 (12.4)	93 (12.7)	134 (12.7)
Perianal/perineal	168 (50.9)	386 (52.9)	554 (52.3)
Thorax/ chest wall	6 (1.8)	29 (4)	35 (3.31)
Abdomen	25 (7.6)	47 (6.4)	72 (6.80)
Limbs	24 (7.2)	28 (3.8)	52 (4.91)
Multiple sites	1 (0.3)	5 (0.7)	6 (0.57)

*Abbreviations* AOR discharge (At own risk discharge), BMI (Body-mass index), DM (diabetes mellitus)

**Fig. 1:**
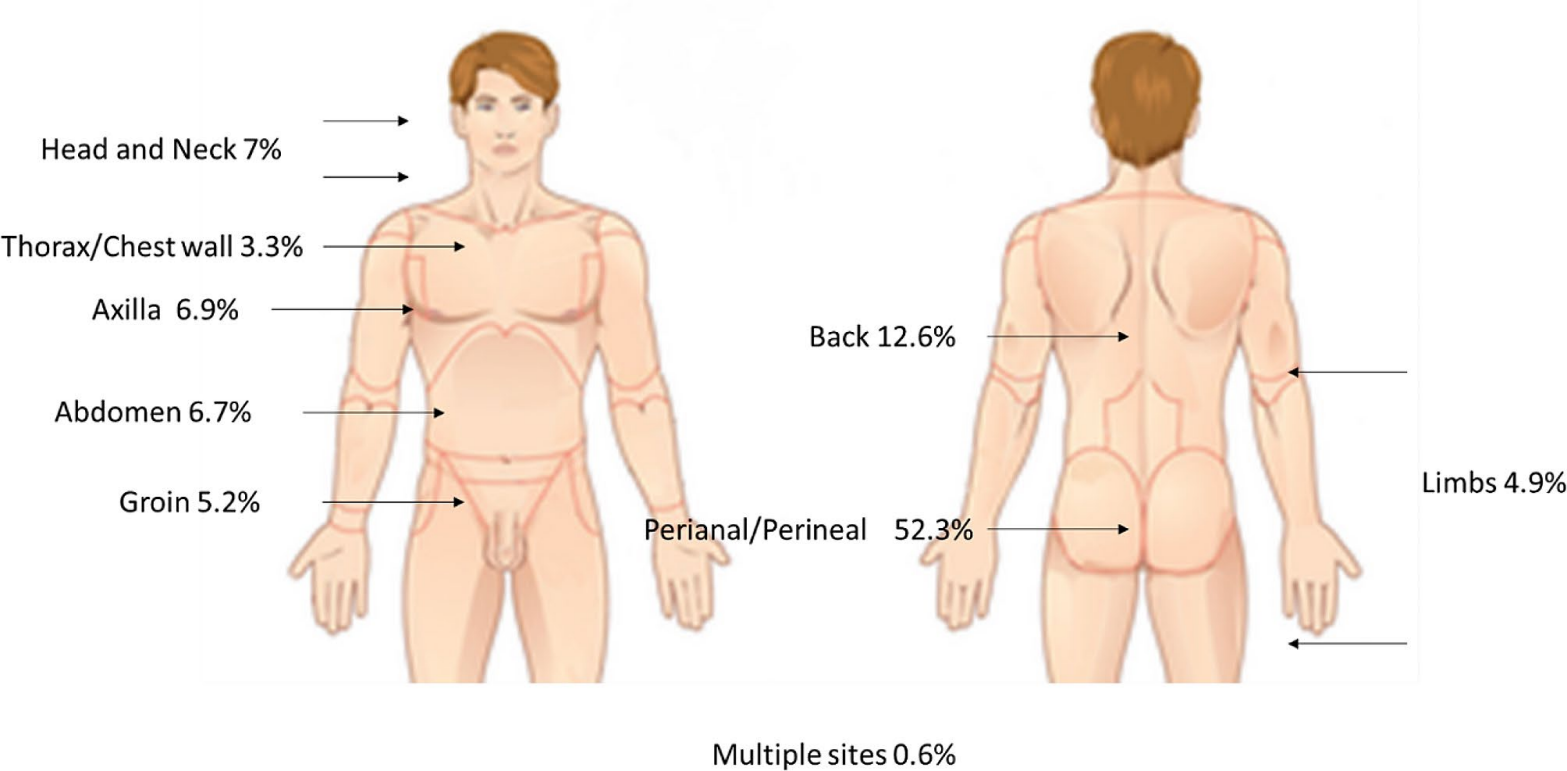
Distribution of STI anatomical locations

Of these patients, 936 (88%) required surgical intervention in the operating theatre and 12% were either treated with antibiotics or underwent a bed-side drainage prior to discharge. The decision for conservative management vs. surgical intervention is a multi-faceted one that was based on clinical assessment of whether drainage was necessary, if the patient’s comorbidities affected the feasibility or safety of surgery and patient preference. The majority of patients were male (63%), under 65 (82%), smokers (74%) with a median BMI of 26, placing them in the overweight category. 31% of patients had pre-existing DM diagnosis with a further 2% newly diagnosed during the index admission. These patients were analysed in the non-diabetic group based on intention to treat basis. Uncomplicated STI admissions were 91% of the cohort. The distribution of STI according to anatomical location is shown in Fig. 1.

Table 2 shows the characteristics and outcomes for patients who underwent surgery for STI according to DM diagnosis.

**Table 2:** Comparison of demographics, surgery type and outcomes between patients with and without DM

	DM (*n* = 330)	Non-DM (*n* = 729)	*P* value (3 sig. fig.)
LOS - days (*N* = 936)	4.46 (5.10)	2.93 (5.09)	< 0.005
BMI kg/m^2^ (*N* = 808)	28.5 (6.43)	26.1 (6.22)	< 0.005
Abscess size – cm^2^ (*N* = 856)	24.5 (36.5)	14.6 (34.7)	0.007
Number of patients underwent wide debridement - % (*N* = 380)	139 [42]	241 [33]	< 0.005
NPWT application - % (*N* = 936)	42 [13]	35 [5]	< 0.005
NPWT duration - days (*N* = 336)	4.6 (10.4)	2.5 (8.8)	0.098
Healing time - days (*N* = 578)	83.6 (141)	66.5 (98.8)	0.061
Fasting or random glucose on admission - mmol/L (*N* = 878)	13.5 (6.96)	6.0 (3.48)	< 0.005
HbA1c on admission - % (*N* = 316)	9.7 (3.37)	4.7 (3.33)	< 0.005
Re-operation same admission (*N* = 936)	8 [2]	4 [0.5]	0.026
Readmission within 30 days (*N* = 43)	15 [4.5]	28 [3.8]	0.869
Recurrence of abscess within 6 months (*N* = 72)	23 [6.9]	49 [6.7]	0.897
Previous abscess within 6 months before this admission (*N* = 114)	36 [14]	78 [14]	0.128

Data are mean (SD), total number [%]. Abbrev: LOS (LOS), BMI (Body-mass Index), NPWT (Negative pressure wound therapy)

Diabetic patients presented with higher BMI (28 vs. 26, *p* < 0.005), larger abscess size (24cm2 vs. 14cm2) and had a longer mean LOS (4.4 vs. 2.9 days, *p* < 0.005). A higher proportion of DM patients required wide debridement as well as application of negative pressure wound therapy (NPWT) (42% vs. 35%, *p* < 0.005). NPWT is generally utilized in patients with larger or more complicated wounds, however there is no existing consensus that guides the need for usage of NPWT currently. The decision for NPWT is one that is made by our team comprising of surgeons and wound specialty nurses based on wound condition, size, depth and patient’s clinical condition and social considerations.

More diabetic patients underwent subsequent re-operation within the same sitting (8 vs. 4). There were no statistically significant differences in re-admission rates within 30 days nor subsequent abscess formation in those followed for 6 months. Re-operation was indicated in patients with worsening or non-resolving STI despite initial surgical drainage as assessed by the surgical team.

### Linear regression models

*Predictors of infection type, LOS, 30-day readmission and recurrence (Tables* *3*, *4*, *5* and *6**)*.

In both uni- and multivariable regression analyses, age (OR = 1.02, *p* < 0.05) and DM (OR$\approx$2.2, *p*<0.05) were independently associated with carbuncle presentation (Table 3).

DM was shown to be a significant independent predictor of LOS (OR 1.17, *p* < 0.0001) as well as age and presence of necrotising infection (Table 4).

**Table 3:** Regression analysis for predictors of infection type

Infection type	Independent variables	OR	95% CI	*p* value	OR	95% CI	*p* value
		Unadjusted			Full		
		Univariate			Multivariable I (Accuracy = 89.03)		
**Cellulitis**							
	**Age**	1.03	(0.994, 1.06)	0.107	1.02	(0.986, 1.06)	0.424
	**Gender**						
	Male (reference)						
	Female	1.27	(0.398, 4.04)	0.687	0.742	(0.202, 2.73)	0.653
	**Smoker status**						
	Non-smoker (reference)						
	Smoker	0.247	(0.032, 1.93)	0.18	0.285	(0.033, 2.5)	0.257
	**BMI**	0.95	(0.85, 1.06)	0.37	0.95	(0.843, 1.07)	0.397
	**Diabetes status**						
	Non-diabetes (reference)						
	Diabetes	1.08	(0.321, 3.62)	0.903	0.858	(0.192, 3.83)	0.841
	**ASA**						
	1 (reference)						
	2	0.462	(0.092, 2.31)	0.347	0.383	(0.061, 2.41)	0.307
	3	1.87	(0.44, 7.97)	0.396	1.09	(0.135, 8.71)	0.939
	4	13.7	(1.16, 162)	0.038	13.7	(0.932, 200)	0.056
**Carbuncle**							
	**Age**	1.02	(1.01, 1.04)	0.006	1.022	(1.00, 1.04)	0.024
	**Gender**						
	Male (reference)						
	Female	0.703	(0.404, 1.22)	0.213	0.941	(0.5, 1.77)	0.85
	**Smoker status**						
	Non-smoker (reference)						
	Smoker	1.24	(0.72, 2.136)	0.438	1.653	(0.872, 3.133)	0.123
	**BMI**	1.00	(0.961, 1.044)	0.943	1.006	(0.961, 1.054)	0.793
	**Diabetes status**						
	Non-diabetes (reference)						
	Diabetes	2.22	(1.34, 3.68)	0.002	2.139	(1.136, 4.027)	0.0185
	**ASA**						
	1 (reference)						
	2	1.17	(0.601, 2.29)	0.64	0.537	(0.219, 1.317)	0.174
	3	1.81	(0.877, 3.75)	0.108	0.544	(0.189, 1.568)	0.259
	4	7.04E-06	(0, INF)	0.986	0	NA	NA

**Table 4:** Regression analysis for of predictors of length of Stay

Independent variables	Estimate	95% CI	*p* value	Estimate	95% CI	*p* value
	Unadjusted			*p* < 0.05 (Adjusted)		
	Univariate			Multivariable II (RMSE = 5.48)		
**Age**	1.006	(1.003, 1.008)	0.0001	1.004	(1.002, 1.007)	0.0003
**Smoker status**						
Non-smoker (reference)						
Smoker	0.975	(0.894, 1.064)	0.575	0.99	(0.91, 1.077)	0.81
**Diabetes status**						
Non-diabetes (reference)						
Diabetes	1.268	(1.171, 1.374)	< 0.0001	1.17	(1.075, 1.273)	0.0003
**Abscess site**						
Abdomen	1.167	(0.996, 1.368)	0.053	1.13	(0.969, 1.318)	0.118
Axilla	0.743	(0.64, 0.864)	< 0.0001	0.786	(0.678, 0.912)	0.001
Back	0.844	(0.751, 0.948)	0.004	0.8	(0.709, 0.904)	0.0003
Groin	0.838	(0.706, 0.994)	0.04	0.805	(0.682, 0.951)	0.01
Head and neck	1.102	(0.944, 1.288)	0.224	1.05	(0.9, 1.225)	0.539
Limbs (upper/lower)	1.332	(1.067, 1.664)	0.0002	1.303	(1.045, 1.624)	0.023
Perianal/perineal (reference)						
Thorax/chest wall	0.787	(0.638, 0.969)	0.024	0.78	(0.637, 0.955)	0.016
**Infection type**						
Abscess (reference)						
Cellulitis	1.263	(0.921, 1.732)	0.169	1.243	(0.911, 1.696)	0.184
Carduncle	0.958	(0.84, 1.092)	0.523	0.933	(0.814, 1.07)	0.321
Necrotizing Fasciitis	12.431	(3.85, 40.14)	< 0.0001	9.819	(3.18, 30.31)	0.0002

In Table 5, a univariable regression yielded significant results for smoker, ASA 3 and having a previous STI within 6 months for predicting readmission within 30-days (OR) of 2.173 (95% CI 1.07–4.41, *p* = 0.036), 2.995 (95% CI 1.096–8.182, *p* = 0.024) and 3.984 (95% CI 1.799–8.824, *p* = 0.002) when compared with non-smoker, ASA 1 and having no abscess within 6 months, respectively. Controlling for the independent predictor variables in Table 5, the multivariable analysis yielded similar pattern of significant associations for having a previous STI within 6 months, however smoker and ASA 3 were no longer significantly associated while a prolonged length of stay. The LOS however became significantly associated with readmission within 30 days (OR = 1.12, 95% CI 1.03–1.22, *p* = 0.042) (Table 5).

**Table 5:** Regression analysis for predictors of 30-day readmission

Independent variables	OR	95% CI	*p* value	OR	95% CI	*p* value
	Unadjusted			Full (Adjusted)		
	Univariate			Multivariable I (AUC = 0.6388, 95% CI 0.4579–0.8196)		
**Age**	1.01	(0.99, 1.03)	0.354	1.004	(0.982, 1.028)	0.73
**Gender**						
Male (reference)						
Female	0.528	(0.23, 1.21)	0.112	0.949	(0.413, 2.18)	0.911
**Smoker status**						
Non-smoker (reference)						
Smoker	2.17	(1.07, 4.41)	0.036	2.16	(0.997, 4.688)	0.072
**BMI**	1.04	(0.988, 1.08)	0.181	1.02	(0.97, 1.063)	0.556
**Diabetes status**						
Non-diabetes (reference)						
Diabetes	1.07	(0.512, 2.22)	0.864	0.498	(0.217, 1.14)	0.132
**Infection type**						
Abscess (reference)						
Cellulitis	0.83	(0.048, 14.3)	0.895	0.278	(0.014, 5.50)	0.438
Carduncle	0.792	(0.213, 2.95)	0.719	1.13	(0.271, 4.67)	0.886
Necrotizing Fascitis	6.91	(0.276, 173)	0.326	4190	(4.83, 3.64E + 06)	0.06
**ASA**						
1 (reference)						
2	1.06	(0.381, 2.93)	0.915	0.661	(0.217, 2.001)	0.516
3	3.00	(1.10, 8.18)	0.024	1.78	(0.479, 6.58)	0.435
4	10.64	(1.39, 81.3)	0.054	5.45	(0.6, 49.6)	0.182
**Abscess site**						
Abdomen	0.553	(0.103, 2.97)	0.453	0.481	(0.094, 2.47)	0.371
Axilla	0.148	(0.009, 2.47)	0.065	0.223	(0.016, 3.07)	0.198
Back	0.434	(0.116, 1.63)	0.17	0.712	(0.186, 2.73)	0.646
Groin	1.01	0.264, 3.88)	0.985	1.17	(0.292, 4.70)	0.838
Head and neck	0.497	(0.093, 2.66)	0.368	0.699	(0.139, 3.5)	0.678
Limbs (upper/lower)	2.67	(0.801, 8.90)	0.146	1.667	(0.452, 6.14)	0.491
Perianal/perineal (reference)						
Thorax/chest wall	0.381	(0.022, 6.49)	0.437	0.439	(0.029, 6.66)	0.528
**Previous abscess within 6 months**	3.98	(1.80, 8.82)	0.002	3.95	(1.72, 9.06)	0.005
**Surgery type**						
Saucerization	0.517	(0.234, 1.14)	0.089	0.597	(0.256, 1.39)	0.274
**VAC**	0.987	(0.264, 3.69)	0.985	0.348	(0.067, 1.82)	0.223
**Reop**	1.27	(0.072, 22.4)	0.876	3.03E-04	(2.72E-07, 3.37E-01)	0.078
**LOS**	1.04	(1.00, 1.07)	0.057	1.12	(1.03, 1.22)	0.042

Having a previous STI within 6 months does increase risk of recurrence (OR = 5.09, 95% CI 2.68–9.67, *p* < 0.001). Undergoing wide debridement puts patients at lower risk of recurrence (OR = 0.411, 95% CI 0.193–0.877, *p* = 0.025), in the multivariable model. (Table 6).

**Table 6:** Logistic regression of predictors of recurrence of STI within 6-months

Independent variables	OR	95% CI	*p* value	OR	95% CI	*p* value
	Unadjusted			Full (Adjusted)		
	Univariate			Multivariable I (AUC = 0.7287, 95% CI 0.6314–0.8261)		
**Age**	1.00	(0.983, 1.02)	0.939	0.995	(0.975, 1.015)	0.646
**Gender**						
Male (reference)						
Female	0.496	(0.253, 0.972)	0.031	0.656	(0.308, 1.397)	0.304
**Smoker status**						
Non-smoker (reference)						
Smoker	2.58	(1.46, 4.56)	0.001	1.916	(0.997, 3.683)	0.065
**BMI**	1.04	(0.995, 1.08)	0.103	1.019	(0.978, 1.062)	0.401
**Diabetes status**						
Non-diabetes (reference)						
Diabetes	0.915	(0.5, 1.68)	0.772	0.602	(0.298, 1.216)	0.182
**Infection type**						
Abdomen (reference)						
Cellulitis	1.67	(0.297, 9.39)	0.584	0.758	(0.106, 5.41)	0.795
Carbuncle	0.489	(0.134, 1.79)	0.232	1.06	(0.27, 4.13)	0.945
Necrotizing Fasciitis	4.27	(0.172, 106)	0.439	2.70	(0.048, 152)	0.648
**ASA**						
1 (reference)						
2	2.10	(0.879, 5.01)	0.075	3.25	(0.902, 11.7)	0.061
3	2.83	(1.11, 7.20)	0.021	5.29	(1.25, 22.3)	0.022
4	8.95	(1.20, 66.6)	0.068	5.52	(0.497, 61.4)	0.217
**Abscess site**						
Abdomen	0.659	(0.176, 2.477)	0.516	0.885	(0.232, 3.38)	0.862
Axilla	1.03	(0.367, 2.87)	0.962	0.899	(0.256, 3.151)	0.872
Back	0.439	(0.143, 1.35)	0.114	0.599	(0.16, 2.25)	0.46
Groin	0.725	(0.192, 2.73)	0.62	1.10	(0.302, 4.04)	0.889
Head and neck	0.865	(0.276, 2.71)	0.8	1.11	(0.297, 4.13)	0.888
Limbs (upper/lower)	3.31	(1.20, 9.11)	0.035	3.26	(1.07, 9.96)	0.061
Perianal/perineal (reference)						
Thorax/chest wall	0.261	(0.015, 4.40)	0.245	0.33	(0.021, 5.05)	0.365
**Previous abscess within 6 months**	5.09	(2.68, 9.67)	5.49E-06	4.07	(1.98, 8.38)	0.0005
**Surgery type**						
I&D (reference)						
Saucerization	0.563	(0.302, 1.05)	0.063	0.411	(0.193, 0.877)	0.025
**VAC**						
0 (reference)						
1	0.586	(0.16, 2.15)	0.385	0.495	(0.12, 2.05)	0.339
**Reop**						
0 (reference)						
1	2.73	(0.462, 16.1)	0.32	2.57E + 00	(0.181, 36.5)	0.525
**LOS**	1.02	(0.986, 1.06)	0.317	1.00	(0.948, 1.06)	0.985

## Discussion

In this study, we have highlighted the significant interplay between DM and presentations for STI. Patients with DM were associated with larger abscesses on presentation including carbuncles, longer LOS, higher use of NPWT as well as re-operation rates. These results highlight the significant healthcare burden that STI’s represent in terms of acute surgical admissions. Furthermore, given that more than 80% of the cohort were under 65 years of age, 74% were smokers and the median BMI was 26, the importance of lifestyle modification for STI prevention cannot be overstated.

DM remains one of the most common chronic illnesses worldwide and bears a significant healthcare burden in terms of morbidity and costs [9]. The prevalence of DM in this cohort of STI patients, 33%, represents a 3-fold higher compared to the 9.5% national prevalence from recent health surveys [15], suggesting diabetic patients are a lot more susceptible to STIs. Therefore, prevention of STI’s as a complication of DM should be an equal healthcare target alongside well-known complications such as foot ulcers. In a local review of foot ulcer disease burden, mean cost per patient was US$3368 for the ulcer alone with costs escalating once patients received local then major amputations [14].

Poor diabetic control is related to a wide range of acute and chronic complications as well as higher rates of hospitalization [13, 16]. Possible mechanisms include impaired neutrophil granulocyte function, antioxidant system and organ dysfunction secondary to vascular complications [7, 15]. In the current study, there were significantly more complicated infections in diabetic patients such as carbuncles (13.6%) as well as larger abscesses on presentation. Our data showed that diabetes was an independent predictor of carbuncle formation (OR 2.22). Early management of these large chronic infections is required to prevent complications such as sepsis, skin grafts and poor healing/scarring [17]. Our data however also highlighted that there was an independent reduction in recurrence with wide debridement. Furthermore, the likelihood of requiring NPWT dressing application suggests a significant increase in costs for the patient and compliance issues may result.

DM was an independent predictor for LOS (OR ). This data is supported by multiple studies in other surgical conditions such as vascular and spinal surgery [18, 19]. These patients have more complex wounds requiring specialised care with greater medical comorbidity complexity. Pre-emptive counselling for the patients in terms of wound management, lifestyle modification (smoking cessation/weight control) as well as efforts to tightly control glucose levels should be mandatory. These should form part of our routine in the management of diabetic STI patients. Furthermore, despite some literature identifying the risks of STI and DM in elderly frail patients, our data highlights the increasing prevalence of DM amongst younger patients (under 65) and the need for counselling earlier in life to prevent the complications of DM [20].

The low incidence of necrotising fasciitis in our cohort is related to the fact that STIs on the limbs are mostly operated upon by the orthopaedic and vascular surgeons in our institution. Nonetheless the identification of DM as a significant risk factor for necrotising STI has been reported previously [21]. Whilst this may have affected the population size studied, the outcomes from this study should be generalizable to STIs on the limbs.

Smoking status was associated with the likelihood of being readmitted or having recurrence of STIs. Smoking increases the risks of microvascular changes leading to premature macrovascular complications in DM patients [22]. There is also a significantly higher proportion of smokers in this cohort than the general population in Singapore (74% vs. 10.1%) [15]. Whilst smoking is known to contribute greatly to the healthcare burden in many ways, this study reinforces the importance of smoking cessation.

Longitudinal data in terms of ongoing glycaemic control, weight and BMI as well as re-presentations for other diabetic complications would be a useful future study. Furthermore, given the poor diabetic control seen in our DM group (mean HbA1c of 9.7%), it indicates the need for secondary prevention for STIs in diabetic patients. A future prospective study incorporating stringent wound management principles as well as lifestyle modification advice would be the next step based upon the current data.

There are some limitations in this study. As a retrospective study, many of the parameters such as type of infection as well as size of the abscess may have varied between clinicians leading to observer and reporting bias. There were also some variables with limited data (e.g. wound healing time) which may have precluded interpretation of results. Other than that, the depth of the abscess was often not recorded, which may have affected the estimation of abscess volume. Patients who were lost to follow up may have presented to other institutions or general practices. Furthermore, the addition of a comparison group (matched-controls) could have alleviated any bias from the study and results. Finally the wound swab data whilst not included in this study, could be useful in the future to assist with difficult to treat infections and antibiotics usage.

## Conclusion

DM is a significant risk factor for STI admission and complications. As the prevalence of DM continues to rise worldwide, the incidence of STIs will represent an ongoing disease burden for the emergency general surgeon as well as a significant cost to patients and institutions alike. Surgeons should consider intensive patient counselling and partnering with primary care providers to reduce the incidence of future STI admissions based upon lifestyle modification and glucose control.

## Data Availability

If required, otherwise not applicable.
